# Secondary-Structure-Dependent Cooperation and Interference Between Peptides of Different Chain Lengths in Antifreeze Activity: Insights from Molecular Dynamics Simulations

**DOI:** 10.3390/foods15122228

**Published:** 2026-06-20

**Authors:** Yuan Yuan, Micholas Dean Smith, Tong Wang

**Affiliations:** 1 Department of Food Science, Institute of Agriculture, University of Tennessee, 2510 River Dr., Knoxville, TN 37996, USA; 2 Department of Biochemistry and Cellular and Molecular Biology, University of Tennessee, Knoxville, TN 37996, USA; msmit316@utk.edu; 3 UT/ORNL Center for Molecular Biophysics, Oak Ridge National Laboratory, Oak Ridge, TN 37831, USA

**Keywords:** antifreeze peptides, secondary structure, chain length, molecular dynamics simulation

## Abstract

Ice recrystallization inhibition (IRI) activity of peptides is influenced by both peptide length and secondary structure; however, whether combinations of peptides with different lengths exhibit cooperative or antagonistic effects remains poorly understood. Using molecular dynamics simulations, this study investigated how secondary structure and chain-length heterogeneity jointly affect the IRI activity of peptide-pair mixtures. For systems containing only β-sheet-rich peptides, mixtures of different chain lengths consistently reduced ice content relative to the corresponding single-peptide systems, suggesting cooperative enhancement of IRI activity. In contrast, individual α-helical peptides showed strong inhibition of ice growth, but this effect was diminished after they were mixed into peptide pairs. Structural analyses suggested that the improved performance of β-sheet mixtures was associated less with the simple preservation of native β-sheet structure than with mixing-induced changes in peptide–peptide coupling and surface exposure. By contrast, helix-containing mixtures retained more of their original local structure in some cases, but this structural retention was not accompanied by improved ice-growth suppression after mixing. Together, these findings suggest that peptide length effects on IRI are not universally synergistic but depend strongly on secondary-structure compatibility.

## 1. Introduction

Previous studies on collagen- and plant-derived protein hydrolysates have shown that peptide molecular size is an important determinant of ice recrystallization inhibition (IRI) activity. In collagen-derived hydrolysates, an optimal molecular weight is typically between 0.6 and 3 kDa, whereas very large peptides (>6–7 kDa) and very small peptides or free amino acids exhibit weak or negligible IRI activity [1,2]. Similarly, antifreeze peptides isolated from silver carp, fish skin, pig skin, and Antarctic krill are frequently enriched in the 0.6–3 kDa range and have shown excellent cryoprotective performance in microbial and cellular systems [3,4]. A similar size-threshold effect was also observed from poly(vinyl alcohol) (PVA), which only shows IRI activity when the degree of polymerization is over 15, because flexible polymers such as PVA require a minimum chain length to propagate binding along the ice surface and reach their full binding potential [5]. However, evidence from soy protein isolate (SPI) hydrolysates and other plant proteins suggests a more complex scenario in which the “optimal size window” can shift and mixtures can outperform isolated fractions. For example, SPI hydrolysates have been reported to exhibit strong IRI in broader and higher-molecular-weight ranges (e.g., 4–14 kDa) [6]; furthermore, peptide mixtures with heterogeneous sizes often display stronger IRI activity than any single size fraction alone [7]. These observations imply that peptide–peptide interactions and collective interfacial behaviors can contribute substantially to IRI and that mixing does not necessarily lead to simple additive effects.

Beyond size, secondary structure is a mechanistically meaningful determinant of IRI activity. Yuan et al. [8] reported a positive correlation between the α-helix structure content of wheat gluten hydrolysates and their IRI activity under different dispersing media. Fu et al. [9] also reported that the enhancement IRI of amyloid protein fibrils from whey protein isolates is related to its β-sheet-rich assembly structure [7]. Previous studies further suggest that molecular conformation and secondary structure can influence antifreeze activity by controlling the spatial presentation of ice-binding residues [10], the organization of amphiphilic or hydration-active motifs [11], and the ordering of interfacial water near ice [12,13]. Thus, peptides with different secondary-structure propensities may differ not only in intrinsic IRI activity, but also in how their functional surfaces are preserved, reorganized, or assembled upon mixing. However, secondary structure alone does not necessarily determine antifreeze activity. For example, studies on winter flounder type I antifreeze protein and its mutants showed that both active and less-active variants remained fully α-helical in water and in ice, indicating that activity differences were more likely related to surface properties than to loss of helical structure itself [14].

Molecular dynamics (MD) simulations provide a powerful tool to probe peptide–water–ice interactions with atomistic resolution and to evaluate how different peptide assemblies modulate ice formation and growth [15]. Although prior computational studies have provided valuable insights into single peptides [16] or idealized antifreeze molecules [17,18], the structural basis by which mixed-peptide assemblies produce either cooperative enhancement or antagonistic interference remains poorly understood.

In this work, representative β-sheet-forming and α-helix-forming peptides were selected because these two structural motifs have both been implicated in antifreeze or ice-interfacial activity, but their behavior in mixed-peptide systems remains insufficiently understood. Within each structural category, peptides with different chain lengths were included to represent chain-length heterogeneity and to allow comparison between single-peptide reference states and mixed-peptide systems. In addition, collagen-derived α-helical peptides were included as an external comparison to examine whether the observed mixing behavior was specific to soybean-derived peptides or reflected a broader structure-dependent trend. We hypothesized that the IRI activity of mixed-peptide systems is not universally synergistic but instead depends on the compatibility between chain-length heterogeneity and secondary-structure preference. Specifically, peptide mixtures with different degrees of structural compatibility were expected to exhibit distinct patterns of secondary-structure retention or redistribution, intermolecular hydrogen bonding, hydration, solvent exposure, and conformational organization, which in turn would be associated with different ice-growth outcomes. To test this hypothesis, we used MD simulations to systematically compare mixing-induced changes in ice growth, secondary structure, intermolecular hydrogen bonding, hydration, solvent exposure, and conformational organization among single-peptide and mixed-peptide systems.

## 2. Methods

### 2.1. Peptide Selection and System Composition

To examine whether mixing peptides of different lengths leads to cooperative or antagonistic effects, peptides were selected to differ primarily in chain length while sharing similar sequence origin and secondary-structure class. Specifically, three β-sheet-forming peptides (21sheet, 40sheet, and 55sheet) and three α-helix-forming peptides (20helix, 37helix, and 57helix) derived from soybean were used to construct simulation systems. Mixed systems were generated by pairing peptides of different lengths within the same secondary-structure class, including 21/40sheet, 21/55sheet, 40/55sheet, 20/37helix, 20/57helix, and 37/57helix. For each peptide pair, simulations were performed for the individual peptides alone, and the corresponding mixed system. Because peptide origin may also influence antifreeze activity, an additional α-helical peptide pair derived from collagen (25helix and 65helix) was included to test whether the observed mixing effects were preserved across peptides from different biological sources with a similar secondary structure. The molecular characteristics of the peptides and simulation systems are summarized in Table 1. Because the number of water molecules and peptide molecular weights differed among systems, the water/MW ratio was used as a simple descriptor of peptide loading. As a sanity check, the average ice percentage at 100 ns was plotted against water/MW, and sensitivity analyses were performed by excluding influential systems (Supplementary Figure S1).

### 2.2. Molecular Dynamics Simulations

#### 2.2.1. System Preparation

Following from our recent work [15], protein sequences were retrieved from the UniProt database (https://www.uniprot.org/), and peptide sequences were generated by tryptic digestion. Initial peptide structures were obtained using PyPept [19] and ColabFold/AlphaFold2 [20,21]. Mixed systems were modeled using Boltz-2 [22] and contained one short peptide chain and one long peptide chain. To maintain comparable chain numbers, single-peptide systems contained two identical chains of the corresponding peptide type. Sodium and chloride ions were added as needed to neutralize the systems and to achieve an ionic strength of 50 mM. All simulations were performed using GROMACS 2021 [23] with the CHARMM36m force field [24] and the TIP5P water model [25] under periodic boundary conditions. TIP5P was selected to maintain consistency with previous antifreeze-peptide molecular dynamics studies from our group and related work, in which this water model was used to qualitatively compare antifreeze behavior among peptide systems [15,26,27,28].

#### 2.2.2. Simulation Protocol

All systems were first energy-minimized using the steepest descent algorithm for 10,000 steps until the maximum force fell below 500 kJ mol^−1^ nm^−1^. Equilibration was then carried out in two stages. First, a 125 ps simulation was performed in the canonical (NVT) ensemble at 265.15 K using the Nosé-Hoover thermostat algorithm for temperature coupling. This was followed by a 125 ps simulation in the isothermal-isobaric (NPT) ensemble at 1 bar using the Parrinello-Rahman barostat algorithm for pressure coupling. Production molecular dynamics simulations were subsequently performed in the NPT ensemble at 265.15 K and 1 bar using the equilibrated structures as starting configurations. Each production trajectory was run for 100 ns with a 2-fs integration time step, and three independent replicates were generated for each system. Long-range electrostatic interactions were calculated using the particle mesh Ewald method. Van der Waals interactions were treated using a force-switch cut-off scheme with smoothing from 1.0 to 1.2 nm. Hydrogen bonds involving hydrogen atoms were constrained using the LINCS algorithm.

### 2.3. Trajectory Analysis

Ice percentages were calculated using the CHILL+ algorithm [29]. For each frame, the fraction of ice-like water molecules was calculated relative to the total number of water molecules and reported as the ice percentage. Secondary structure was assigned using the Dictionary for Secondary Structure Prediction (DSSP). To facilitate comparison across systems, DSSP states were grouped into three classes: helix (α-helix, 3_10_-helix, and π-helix), sheet (extended β-strand and β-bridge), and coil (all remaining DSSP states). For each frame, the fraction of residues in each class was calculated by dividing the number of residues in that class by the total number of peptide residues and averaging over the specified time windows. Hydrogen bonds were analyzed using GROMACS. Peptide–peptide and peptide–water hydrogen bonds were quantified as observed donor–acceptor interactions according to the default geometric criteria of the software. Hydrogen-bond counts were normalized by the number of possible donor–acceptor pairs to facilitate comparison among systems of different sizes. For single-peptide systems, solvent-accessible surface area (SASA), root-mean-square deviation (RMSD, calculated with respect to the first frame of the production trajectory) and radius of gyration (Rg) were calculated separately for each of the two identical chains and then averaged to obtain the value for that peptide type. For mixed systems, the same metrics were calculated separately for the short and long peptide chains.

### 2.4. Statistical Analysis

All simulations were performed in triplicate. Results are presented as mean ± standard deviation unless otherwise specified. For time-dependent analyses, the mean trajectory was plotted with the standard deviation across replicates. For time-window analyses, each replicate trajectory was first averaged over the specified analysis window, such as the final 20 ns or the last 50 ns, and the replicate-level averages were then used to calculate the mean and standard deviation. The relationship between water/MW ratio and ice percentage was evaluated as an exploratory linear regression analysis, and the coefficient of determination (R^2^) was reported. Figures were generated by using the Matplotlib library, version 3.7.2.

## 3. Results and Discussion

### 3.1. Ice Percentage Content in Single and Mixed-Peptide Systems

**Figure 1:**
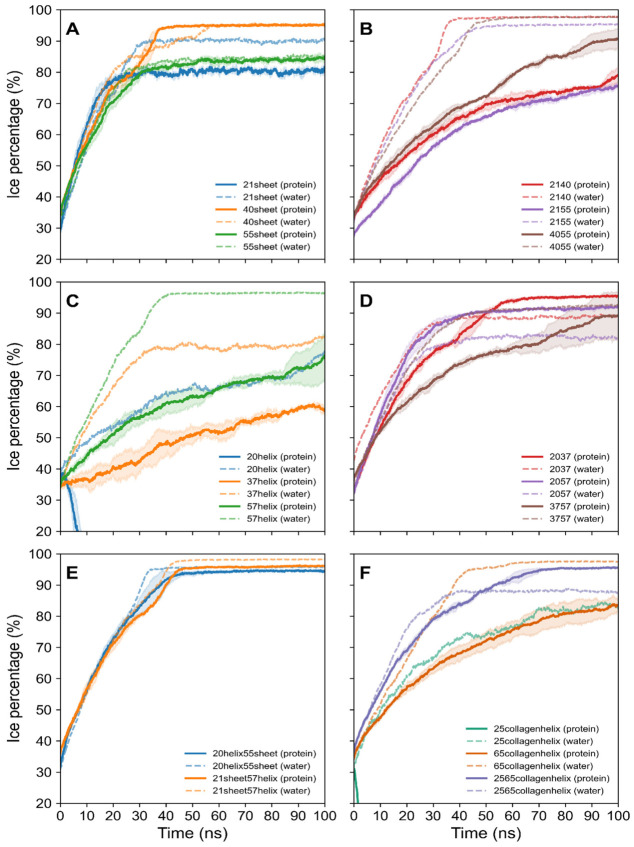
Ice percentage evolution in MD simulations for single peptides and peptide mixtures over 100 ns (nanoseconds) of simulation time. (**A**) Single-sheet peptides from soybean. (**B**) Mixed-sheet peptide pairs from soybean. (**C**) Single-helix peptides from soybean. (**D**) Mixed-helix peptide pairs from soybean. (**E**) Cross-structure mixtures from soybean (20helix + 55sheet and 21sheet + 57helix). (**F**) Cross-source validation using collagen-derived helix peptides. Solid lines represent peptide-containing systems (“peptide”), and dashed lines represent the corresponding water-only controls prepared under identical conditions. Curves show the mean, and shaded regions indicate ±SD from three independent replicates.

The time-dependent evolution of ice percentage is presented in Figure 1 for both single-peptide and mixed-peptide systems, together with the corresponding water-only controls.

Among the individual β-sheet peptides, 21sheet, 40sheet, and 55sheet showed limited ability to suppress ice growth, as their ice-percentage profiles remained close to, or only slightly below, those of the water-only controls (Figure 1A). In contrast, the individual α-helical peptides derived from soybean (20helix, 37helix, and 57helix) and collagen (25helix) displayed much stronger inhibition of ice growth, with substantially lower ice percentages than their corresponding controls throughout the simulations (Figure 1C,F). This observation is consistent with previous studies suggesting that more ordered peptide conformations, particularly α-helical structures, may be associated with stronger antifreeze-related activity [8,30,31]. Interestingly, the short single-helix peptides, 20helix and 25helixcollagen, showed net reductions in ice-like water content under the original simulation conditions. Although short helical peptides have previously been associated with antifreeze-related activity [32], the present simulations alone cannot establish a strong thermodynamic ice-melting effect or freezing-point depression. To further examine whether this melting-like behavior was robust or sensitive to the simulation setup, additional large-box simulations were performed for 20helix and 25helixcollagen (Figure S3). For 20helix, the rapid decrease in ice-like water content observed in the normal box was not reproduced in the large-box simulation; instead, the larger system showed an increase in ice-like water content, indicating freezing under the larger-box condition. For 25helixcollagen, the large-box simulation still showed a decrease in ice-like water content, but the decrease occurred more slowly than in the normal box. These results suggest that the melting-like behavior of the two short helical peptides was sensitive to system size and/or effective peptide concentration. Similar finite-size or concentration-related effects may also influence some of the other systems, but a systematic investigation of these effects is beyond the scope of the present work.

A markedly different pattern was observed after peptide mixing. For the sheet systems, mixtures of different chain lengths (21/40sheet, 21/55sheet, and 40/55sheet) consistently produced lower ice percentages than the corresponding single-sheet peptides over the simulation time (Figure 1B), indicating slower ice growth upon mixing. In contrast, mixing helical peptides led to the opposite outcome. The helix–helix combinations (20/37helix, 20/57helix, 37/57helix, and 25/65collagenhelix) showed substantially higher ice content than their individual components, with several mixed systems approaching the behavior of the water-only controls (Figure 1D,F). A similar loss of activity was also observed in the cross-structure mixtures (20helix + 55sheet and 21sheet + 57helix), whose ice percentage were similar to those of the controls (Figure 1E). These results suggest that peptide mixing does not uniformly improve antifreeze activity; rather, the effect of mixing depends strongly on the secondary structure identities of the peptide partners. Within the present dataset, β-sheet mixtures showed lower ice percentages after mixing, whereas helix–helix and helix–sheet mixtures showed reduced antifreeze activity. Because peptide loading varied among systems, a sanity-check analysis was performed to examine whether the observed differences in ice growth inhibition could be explained simply by peptide loading (water/MW ratio) (Table 1). When the average ice percentage at 100 ns was plotted against water/MW for all systems, a moderate negative relationship was observed (R^2^ = 0.422; Figure S1A). However, this trend was strongly influenced by two unusual helical systems—20helix and 25helixcollagen—both of which showed ice-melting behavior. Excluding 20helix or 25helixcollagen reduced the correlation to R^2^ = 0.287 and 0.323, respectively, and excluding both reduced it further to R^2^ = 0.097 (Figure S1B–D). These results indicate that peptide loading may contribute to the overall trend, but does not consistently explain the system-dependent differences in ice growth inhibition. Instead, peptide-specific structural features and intermolecular organization appear to play a more important role.

**Figure 2:**
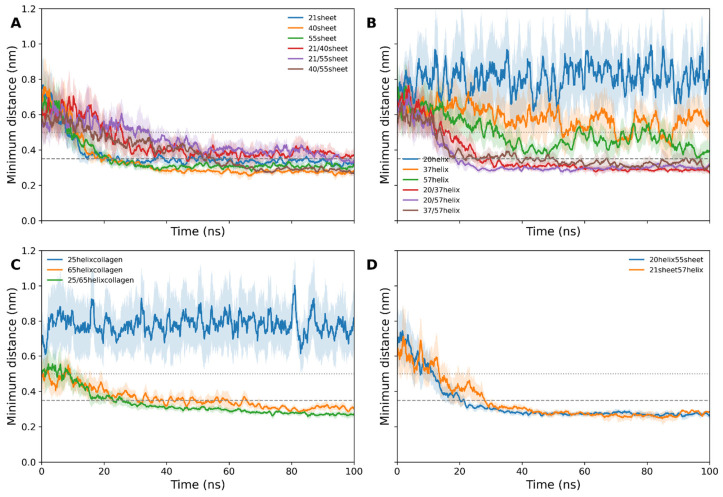
Time-dependent minimum distance between peptides and ice-like water molecules during MD simulations. (**A**) Sheet mixtures: 21/40sheet, 21/55sheet, and 40/55sheet. (**B**) Helical mixtures: 20/37helix, 20/57helix, and 37/57helix. (**C**) Collagen-derived helical mixture: 25/65collagenhelix. (**D**) Cross-structure mixtures from soybean: 20helix55sheet and 21sheet57helix. The horizontal dashed line at 0.35 nm indicates hydrogen-bond-scale proximity to the ice surface, while the dotted line at 0.50 nm marks a more relaxed near-surface reference distance.

Because the short single helical systems showed unusually strong decreases in ice-like water content, we further examined whether this behavior was associated with direct peptide access to the ice surface. For most systems, the closest peptide–ice distance decreased to approximately 0.30–0.35 nm, indicating hydrogen-bond-scale proximity to the ice surface (Figure 2). The main exceptions were 20helix and 25helixcollagen, which maintained much larger apparent minimum distances and showed nearly no peptide–ice contacts (Figure S4). Because these two systems also showed a reduction in ice-like water content, the larger apparent peptide–ice distances likely reflect the reduced or unstable amount of remaining ice-like water in these melting-like simulations, rather than simply indicating weak intrinsic peptide–ice affinity. Across the remaining systems, peptide–ice contact number did not show a simple relationship with ice-growth suppression, suggesting that direct ice contact was not sufficient to explain antifreeze-related performance. Therefore, subsequent analyses focused on secondary structure, peptide–peptide coupling, hydration, solvent exposure, and conformational organization.

### 3.2. Secondary Structure Dynamics in Mixed Systems

**Figure 3:**
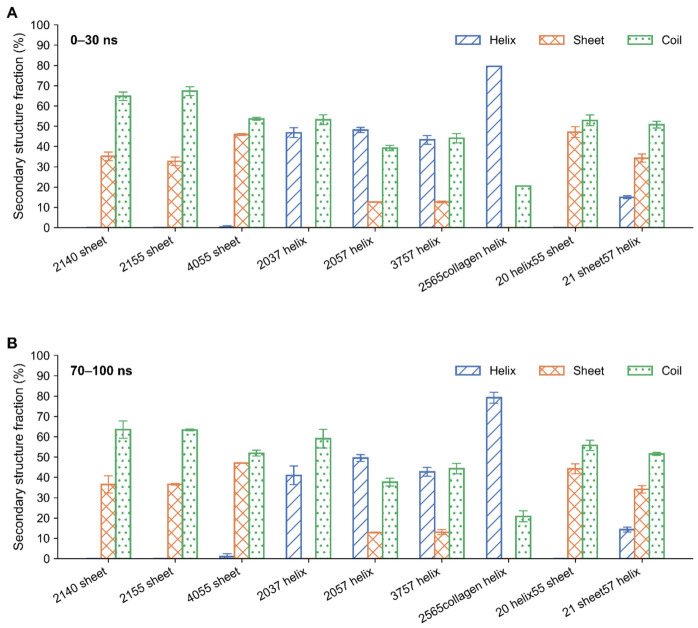
Secondary-structure composition of mixed-peptide systems during the early and late simulation stages: (**A**) 0–30 ns and (**B**) 70–100 ns.

To investigate whether peptide mixing altered secondary-structure behavior in the mixed systems, the overall secondary-structure compositions of the combined systems were first compared during the early (0–30 ns) and late (70–100 ns) simulation periods. At the whole-system level, only minor differences were observed between the early and late stages (Figure 3), indicating that the total secondary-structure composition of most mixed systems remained relatively stable.

**Figure 4:**
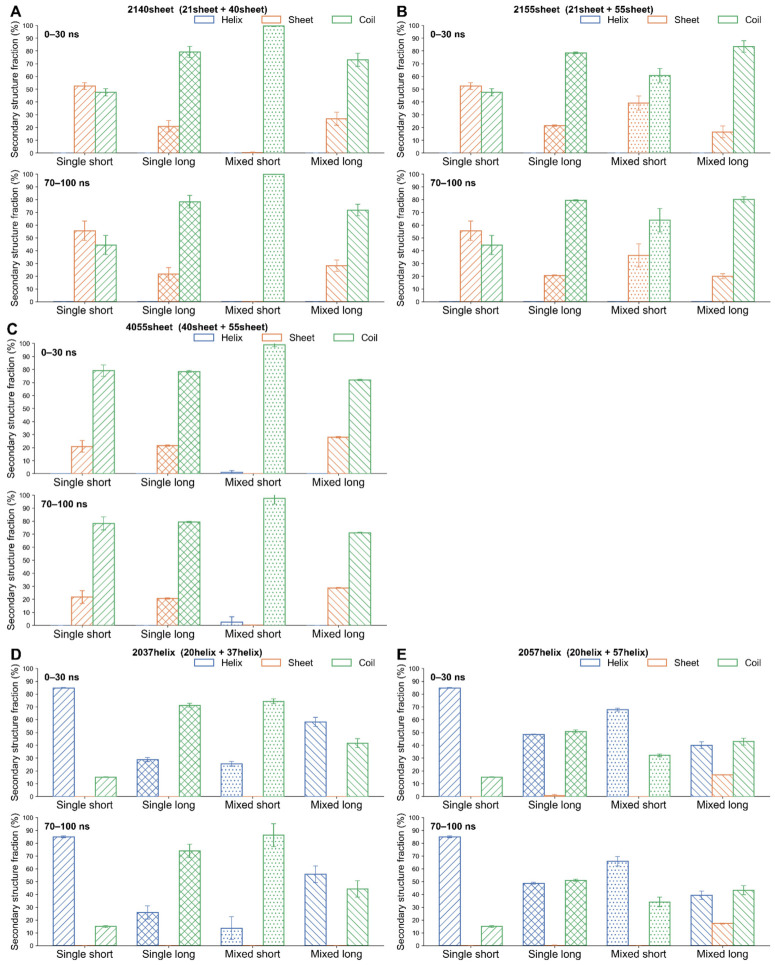

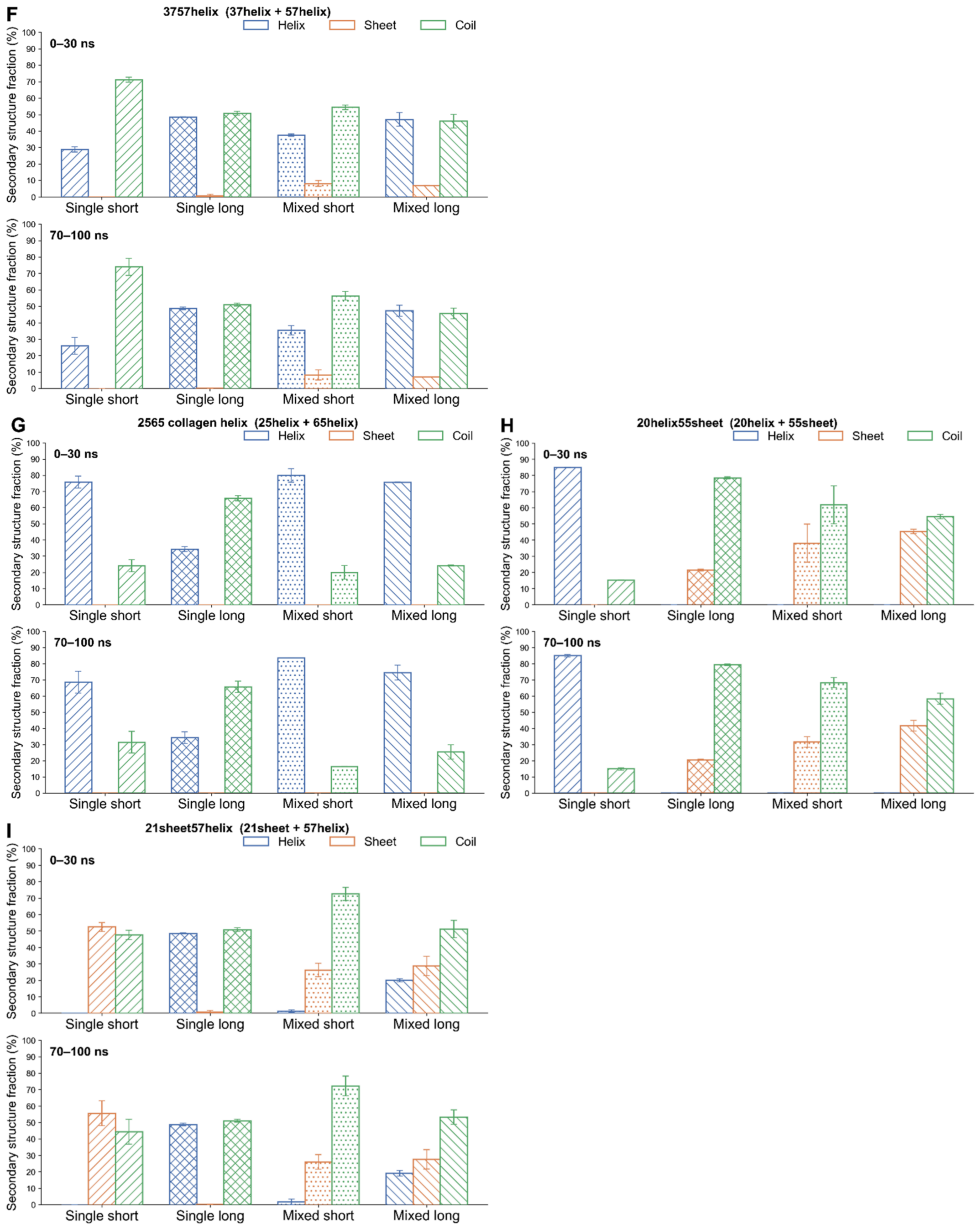
Secondary structure compositions of peptides in single and mixed systems. (**A**–**C**) Sheet mixtures: 21/40sheet, 21/55sheet, and 40/55sheet. (**D**–**F**) Helical mixtures: 20/37helix, 20/57helix, and 37/57helix. (**G**) Collagen-derived helical mixture: 25/65collagenhelix. (**H**,**I**) Cross-structure mixtures from soybean: 20helix55sheet and 21sheet57helix. For each mixed system (titles above each block), secondary structure was computed separately for the short and long peptides in the corresponding single-peptide simulations (“Single short/Single long”) and within the mixed-peptide simulations (“Mixed short/Mixed long”).

However, this stability did not necessarily mean that both peptide components responded similarly to mixing. To resolve the structural contribution of each component, the short and long peptides were analyzed separately and compared with their corresponding single-peptide states (Figure 4).

For sheet–sheet combinations, the most prominent change after mixing was a reduction in β-sheet content in the short peptide, accompanied by an increase in coil fraction. For example, in the 21/40sheet system, the β-sheet fraction of the short peptide decreased from approximately 50% in the single-peptide state to nearly 0% after mixing, while the coil fraction increased from 50% to 100%. A similar trend was observed in the 40/55sheet system, where the mixed short peptide became more coil-rich than its corresponding single-peptide state. In contrast, the long peptide was comparatively less affected and showed smaller changes in β-sheet content between the single and mixed states. These observations suggest that sheet–sheet mixing was not simply characterized by preservation of the original β-sheet-rich state of the short peptide but instead was associated with local secondary-structure reorganization. This trend appeared within the first 30 ns and remained generally similar during the final 30 ns, suggesting that the main secondary-structure adjustment occurred early and persisted over the simulation period. However, the helix–helix combinations displayed a different structural response. Both short and long peptides generally retained a measurable fraction of helix structure after mixing, rather than undergoing the extensive rigid-to-coil transition observed in the sheet mixtures. This behavior was especially evident in 25/65collagenhelix, in which both the mixed short and mixed long peptides maintained relatively high helix content. However, retention of helical structure did not necessarily correspond to stronger ice-content suppression, indicating that secondary-structure preservation alone was insufficient to explain antifreeze performance in mixed systems. The helix–sheet combinations showed the largest component-specific structural changes upon mixing. In these systems, both components, especially the short peptide, lost much of their original dominant secondary structure. For example, in the 20helix55sheet system, the helix content of the short peptide decreased from ~80% in the single-component system to nearly zero after mixing, while the sheet content of the long peptide increased from ~25% to ~50%. In the 21sheet57helix system, the helix content of the helix-rich peptide decreased from ~50% to ~20%. Consistently, across helix–sheet combinations, the helix-rich peptide became less helical, while the sheet-rich partner exhibited a redistribution of its secondary structure populations. These results suggest that helix–sheet mixing substantially perturbed the preferred local conformations of both peptide components and may reflect reduced local structural compatibility between helix-type and sheet-type peptides after mixing.

These results show that different peptide mixtures follow different local adaptation pathways after mixing. In the sheet mixtures, partial loss of β-sheet character, especially in the short peptide, did not prevent improved ice-growth suppression. This suggests that IRI-related behavior in mixed systems may depend not only on the abundance of a single secondary-structure type, but also on how structural flexibility and intermolecular interactions are reorganized after mixing. In contrast, helix-containing mixtures often retained more of their original helical structure, but this structural retention was not accompanied by improved ice-growth suppression. This discrepancy between structural retention and ice-growth suppression suggests that secondary-structure content alone cannot fully explain the mixed-system behavior, and other intermolecular interactions must also be considered.

### 3.3. Hydrogen-Bond Reorganization in Mixed Systems

**Figure 5:**
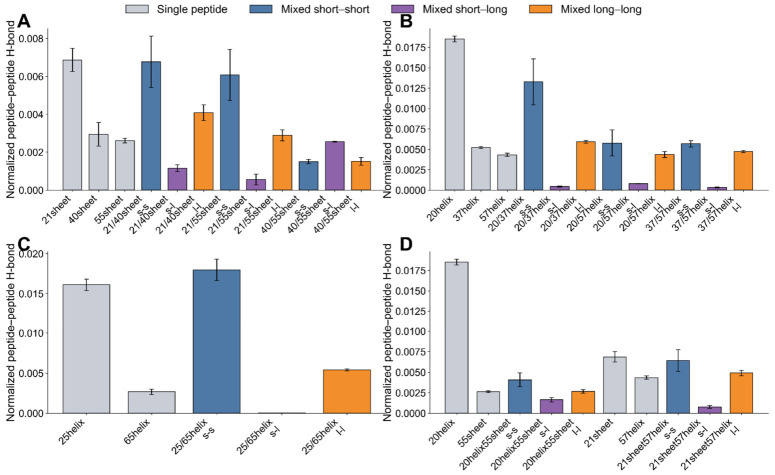
Normalized peptide-peptide hydrogen bonds during the last 50 ns of the simulations. (**A**) Sheet mixtures: 2140sheet, 2155sheet, and 4055sheet. (**B**) Helical mixtures: 2037helix, 2057helix, and 3757helix. (**C**) Collagen-derived helical mixture: 2565collagenhelix. (**D**) Cross-structure mixtures from soybean: 20helix55sheet and 21sheet57helix.

The peptide–peptide hydrogen-bond analysis is shown in Figure 5. The short–long hydrogen bonding remained higher in the sheet–sheet combinations than in the helix–helix or helix–sheet mixtures. This observation indicates that sheet pairing was associated with stronger direct intermolecular coupling between the two peptide components. This suggests that the advantage of sheet mixtures does not arise simply from preserving the original conformation of each peptide, but rather from forming a more connected interaction pattern after mixing. Previous studies have shown that intermolecular hydrogen bonding can contribute to antifreeze-related peptide assembly. For example, molecular simulations of putative antifreeze cyclic peptides studied by Brotzakis et al. [33] showed that intermolecular Thr side-chain hydrogen bonds contributed to the stability of larger assemblies, while Kim et al. [34] reported that self-assembled homo-oligopeptide nanostructures exhibited enhanced ice growth inhibition. Increased peptide–peptide hydrogen bonding may therefore contribute to antifreeze activity indirectly by stabilizing peptide–peptide contacts or maintaining spatial arrangements that are compatible with ice-growth suppression. However, helix–helix mixtures, despite retaining more of their original helical structure in some cases, did not establish equally strong short–long hydrogen bonding. The helix–sheet mixtures showed both stronger secondary-structure perturbation and weaker intermolecular coupling.

**Figure 6:**
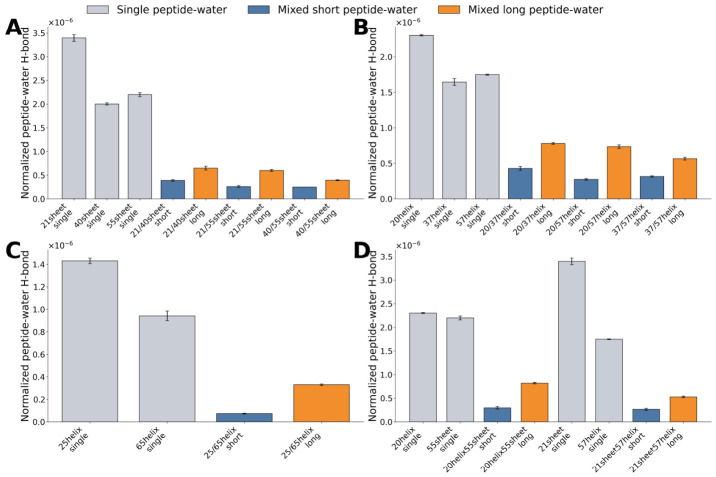
Normalized peptide-water hydrogen bonds during the last 50 ns of the simulations. (**A**) Sheet mixtures: 21/40sheet, 21/55sheet, and 40/55sheet. (**B**) Helical mixtures: 20/37helix, 20/57helix, and 37/57helix. (**C**) Collagen-derived helical mixture: 25/65collagenhelix. (**D**) Cross-structure mixtures from soybean: 20helix55sheet and 21sheet57helix.

Peptide–water hydrogen bonding (Figure 6) is often considered relevant to antifreeze activity because it may help stabilize ordered interfacial hydration [35], support persistent water-mediated contacts at the peptide surface [36], and perturb local water dynamics near the growing ice interface [37]. In the present study, however, peptide–water hydrogen bonding decreased markedly in both sheet and helix mixtures relative to the corresponding single-peptide states, indicating that direct peptide hydration was generally reduced after mixing. This trend suggests that the absolute number of peptide–water hydrogen bonds may not fully explain the antifreeze activity among the mixed systems. Instead, differences in hydration organization, hydrogen-bond persistence, and the extent to which hydration loss is accompanied by favorable intermolecular association may be more important [38]. In the sheet mixtures, reduced peptide hydration was accompanied by preserved or enhanced peptide–peptide hydrogen bonding, which is consistent with partial compensation through stronger intermolecular coupling. In the helix mixtures, by contrast, peptide–water and peptide–peptide hydrogen bonding decreased simultaneously, suggesting hydration loss without a corresponding increase in short–long peptide coupling. Thus, these results suggest that hydration loss alone is not sufficient to explain activity, but antifreeze-related performance appears to depend on whether changes in hydration occur together with interaction patterns that are related with ice-growth suppression.

### 3.4. Surface Exposure and Conformational Organization of Mixed Systems

**Figure 7:**
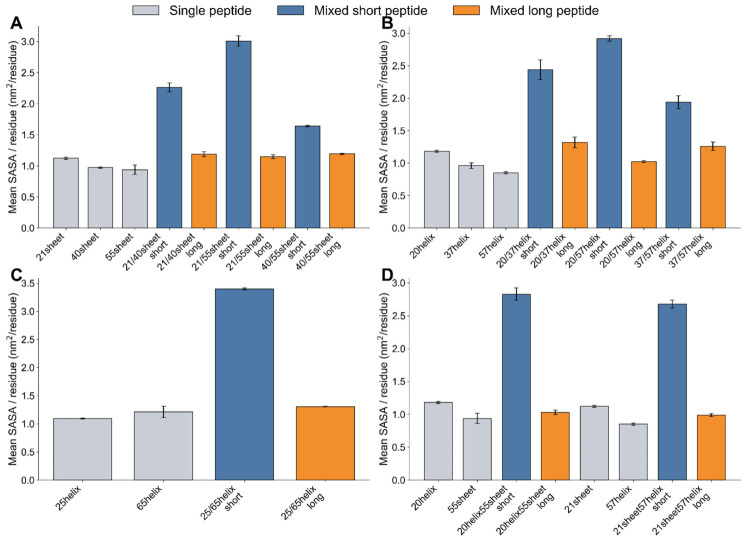
Solvent-accessible surface area (SASA) of short and long peptides during the last 50 ns of the simulations in single and mixed states. (**A**) Sheet mixtures: 2140sheet, 2155sheet, and 4055sheet. (**B**) Helical mixtures: 2037helix, 2057helix, and 3757helix. (**C**) Collagen-derived helical mixture: 2565collagenhelix. (**D**) Cross-structure mixtures from soybean: 20helix55sheet and 21sheet57helix.

To further determine whether reduced peptide hydration reflected global solvent exposure or component-specific surface redistribution, SASA was determined (Figure 7). Among all mixed systems, the SASA per residue increased strongly for the short peptide, whereas the long peptide showed only modest changes relative to the corresponding single-peptide state. This solvent exposure behavior indicates that mixing did not simply bury either peptide within a collapsed aggregate but promoted increased surface exposure of the short peptide in the mixed pairs. Importantly, increased surface exposure alone did not explain antifreeze activity, because both sheet and helix mixtures displayed increased short-peptide SASA. Previous studies have suggested that greater exposure of ice-binding-relevant surfaces is associated with increased antifreeze activity [12,39]. Therefore, higher SASA combined with productive intermolecular organization may be more favorable for stronger antifreeze activity. In sheet mixtures, increased short-peptide exposure occurred together with preserved or enhanced peptide–peptide hydrogen bonding, suggesting that the exposed short peptide remained involved in intermolecular interactions. In helix mixtures, however, similarly increased short-peptide SASA was not accompanied by stronger short–long coupling. This suggests that increased surface exposure may be insufficient for improved ice-growth suppression unless it occurs together with suitable intermolecular interactions.

**Figure 8:**
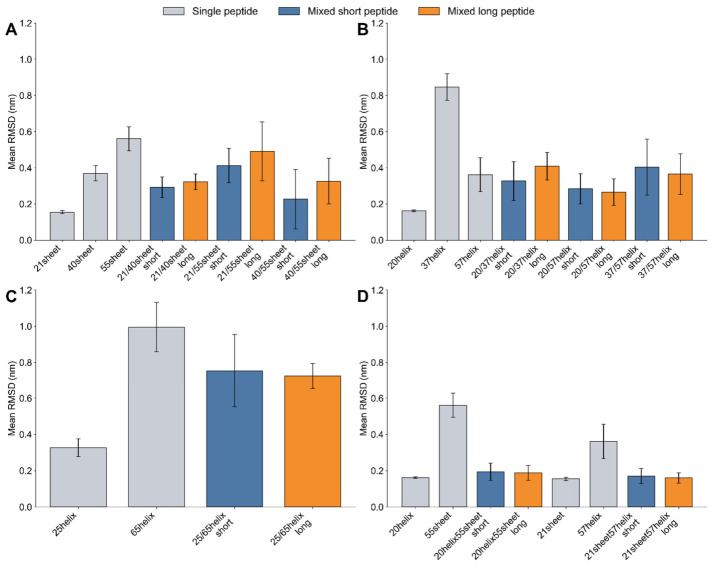
Backbone root-mean-square deviation (RMSD) of short and long peptides during the last 50 ns of the simulations in single and mixed states. (**A**) Sheet mixtures: 2140sheet, 2155sheet, and 4055sheet. (**B**) Helical mixtures: 2037helix, 2057helix, and 3757helix. (**C**) Collagen-derived helical mixture: 2565collagenhelix. (**D**) Cross-structure mixtures from soybean: 20helix55sheet and 21sheet57helix.

**Figure 9:**
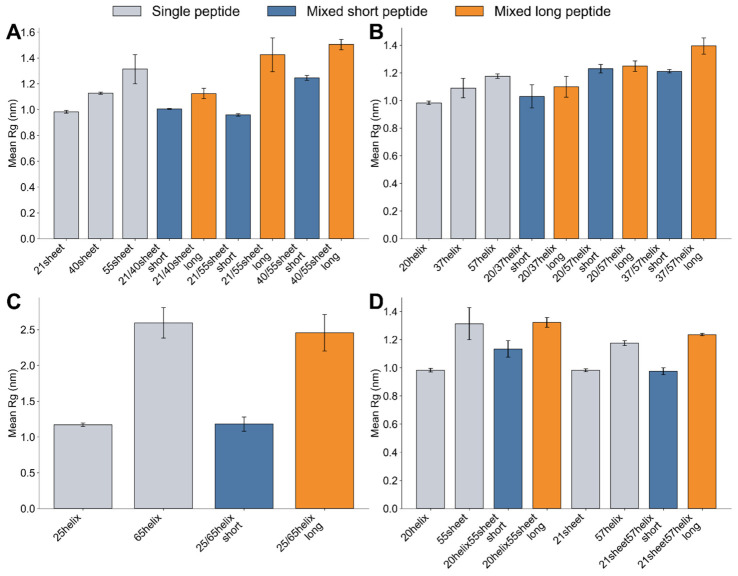
Radius of gyration (Rg) of short and long peptides during the last 50 ns of the simulations in single and mixed states. (**A**) Sheet mixtures: 2140sheet, 2155sheet, and 4055sheet. (**B**) Helical mixtures: 2037helix, 2057helix, and 3757helix. (**C**) Collagen-derived helical mixture: 2565collagenhelix. (**D**) Cross-structure mixtures from soybean: 20helix55sheet and 21sheet57helix.

To further evaluate whether the mixed-state surface redistribution was accompanied by conformational rearrangement, RMSD (Figure 8) and Rg (Figure 9) were analyzed. In most mixtures, the short peptide showed higher RMSD after mixing, whereas the long peptide showed lower RMSD relative to the corresponding single-peptide state. In contrast, the Rg values of both peptides changed only modestly after mixing, indicating that these rearrangements were not associated with major global changes in chain compactness. In some antifreeze-peptide systems, lower RMSD and more stable Rg have been associated with higher activity because they may reflect greater structural stability, reduced flexibility, and improved stability of favorable ice-binding sites [40]. However, this relationship is not universal, as other active systems displaying conformational flexibility and structural diversity are more beneficial for IRI activity [26]. Therefore, RMSD and Rg alone are insufficient to explain antifreeze activity, and they should be interpreted together with hydrogen bonding and SASA data to determine whether conformational reorganization is effective or ineffective. In the sheet mixtures, the increase in short-peptide RMSD, together with relatively stable Rg value in several cases, is consistent with controlled conformational adaptation rather than simple collapse, especially when considered alongside stronger short–long hydrogen bonding and altered surface exposure. In contrast, the helix mixtures did not show the same combination of structural and interaction features. Although some mixed-helix systems exhibited lower RMSD than the corresponding highly fluctuating single helices, this apparent stabilization was not accompanied by stronger hydrogen bonding or improved antifreeze activity. Their Rg values also did not indicate a clear transition toward a more efficiently organized assembly. Taken together, the combined RMSD, Rg, hydrogen-bond, and SASA results suggest that helix mixtures underwent ineffective rearrangement, structural relaxation, or constrained coexistence rather than functionally favorable coexistence.

## 4. Conclusions

Overall, this study shows that the antifreeze activity of mixed-peptide systems is governed by many factors, such as secondary structure and interaction with water/ice molecules, rather than by a simple additive effect of individual peptides. Although some short helical peptides showed strong antifreeze activity as single components, this activity was not retained after mixing, suggesting that helix-containing combinations may alter the intermolecular and interfacial interactions associated with effective antifreeze behavior. In contrast, β-sheet mixtures showed mixing-induced structural and interaction changes, including enhanced peptide–peptide coupling and favorable surface redistribution, which may contribute to their improved ice-growth suppression despite partial loss of native sheet structure. These findings indicate that peptide-based antifreeze systems should be designed not only by selecting individually active peptides, but also by considering whether peptide mixing promotes productive intermolecular association and peptide–ice/water interactions. This work provides a mechanistic framework for designing food-derived peptide mixtures with improved cryoprotective performance.

## Figures and Tables

**Table 1:** Molecular characteristics and simulation box parameters of peptides and peptide mixtures.

Secondary Structure	Sample ID	Protein Source	Total MW (Da)	Box Size (nm)	Number of Water Molecules	Water/MW
Sheet	21sheet	SoybeanP25974	2509	4.7 × 6.6 × 7.0	6651	2.65
	40sheet	SoybeanP11827	5179	6.9 × 5.8 × 9.0	11,346	2.19
	55sheet	SoybeanP25974	6162	8.6 × 5.1 × 9.4	12,369	2.01
	21/55sheet	Soybean	8671	11.0 × 7.4 × 9.7	21,748	2.51
	21/40sheet	Soybean	7688	7.6 × 7.9 × 9.9	18,264	2.38
	40/55sheet	Soybean	11,341	8.4 × 7.9 × 12.9	26,362	2.32
Helix	20helix	SoybeanP11826	2334	5.5 × 5.2 × 9.1	7017	3.00
	37helix	SoybeanP04776	3994	6.1 × 6.4 × 9.4	11,367	2.85
	57helix	SoybeanP0DO15	6562	6.8 × 7.1 × 9.4	14,237	2.17
	20/37helix	Soybean	6328	6.9 × 7.2 × 9.9	15,345	2.42
	20/57helix	Soybean	8896	8.5 × 7.9 × 9.7	20,576	2.31
	37/57helix	Soybean	10,556	8.4 × 7.9 × 11.6	23,989	2.27
Helix (other source pair)	25helixcollagen	CollagenP25067	2593	5.9 × 5.6 × 7.1	7612	2.94
	65helixcollagen	CollagenQ14993	7614	10.7 × 5.0 × 12.4	20,693	2.72
	25 + 65collagen	Collagen	10,207	6.9 × 12.2 × 11.1	29,728	2.91
Helix + sheet	20helix55sheet	Soybean	8496	7.6 × 7.9 × 9.9	19,114	2.25
	21sheet57helix	Soybean	9071	7.7 × 7.9 × 11.2	21,570	2.38

## Data Availability

The original contributions presented in the study are included in the article/Supplementary Material, further inquiries can be directed to the corresponding authors.

## Supplementary Materials

The following supporting information can be downloaded at: https://www.mdpi.com/article/10.3390/foods15122228/s1, Figure S1: Sanity-check analysis of the relationship between peptide loading and ice suppression. The average ice percentage at 100 ns was plotted against the water/MW ratio for (A) all systems, (B) all systems excluding 20helix, (C) all systems excluding 25helixcollagen, and (D) all systems excluding both 20helix and 25helixcollagen; Figure S2: Final 20 ns block-averaged ice percentage of peptide-containing systems and water controls; Figure S3: Comparison of ice percentage in normal-box and large-box simulations for 20helix and 25helixcollagen at 265.15 K; Figure S4: Time-dependent peptide–ice contact number during MD simulations. (A) Sheet mixtures: 2140sheet, 2155sheet, and 4055sheet. (B) Helical mixtures: 2037helix, 2057helix, and 3757helix. (C) Collagen-derived helical mixture: 2565collagenhelix. (D) Cross-structure mixtures from soybean: 20helix55sheet and 21sheet57helix.
