# Supplementary figures and images for: Secondary-Structure-Dependent Cooperation and Interference Between Peptides of Different Chain Lengths in Antifreeze Activity: Insights from Molecular Dynamics Simulations
Source: Foods. 2026 Jun 20;15(12):2228. doi: 10.3390/foods15122228 (PMC13298197; doi:10.3390/foods15122228)

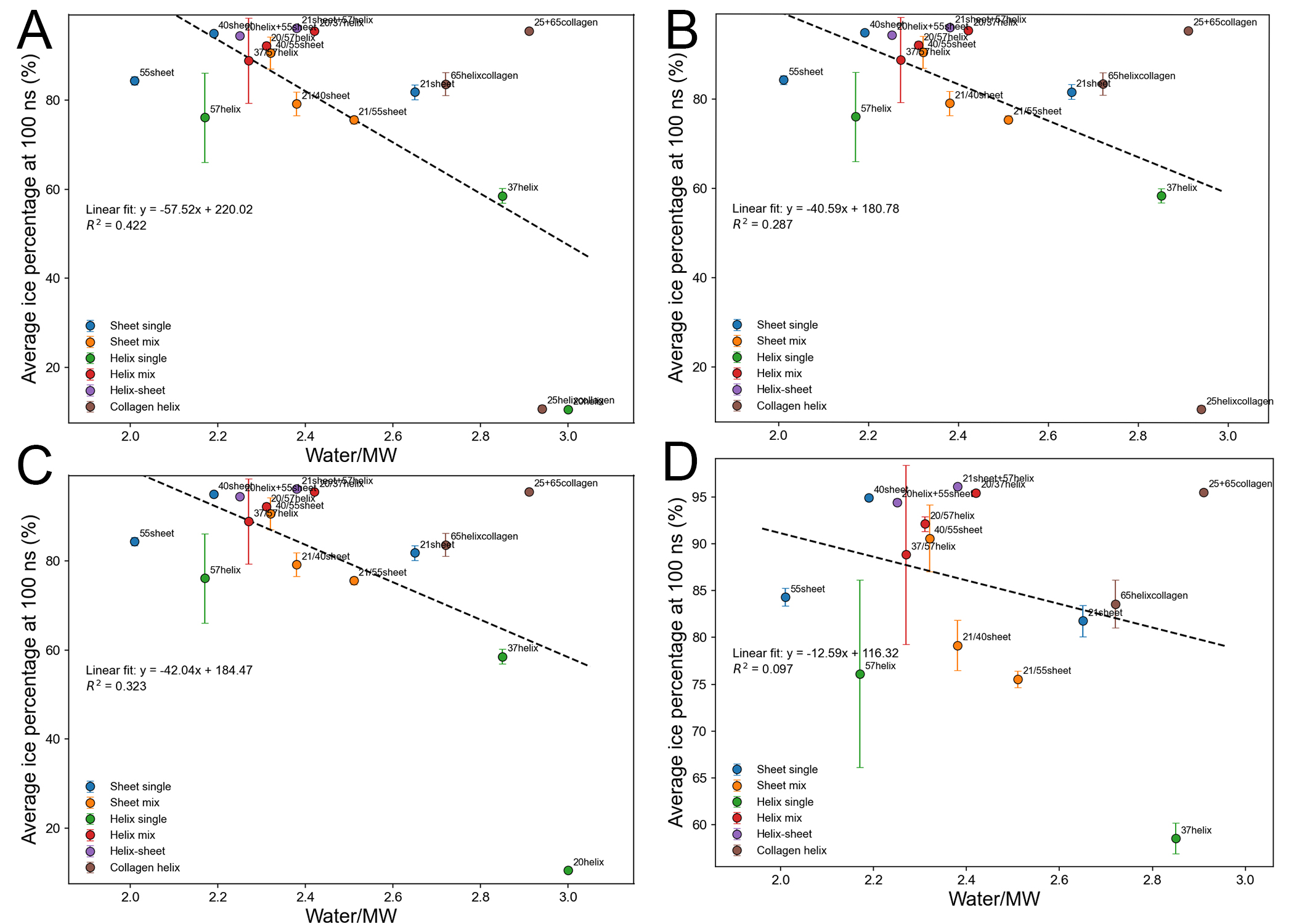

Supplement: Supplementary file 1 [file foods-15-02228-s001.zip › FigureS1.jpg]

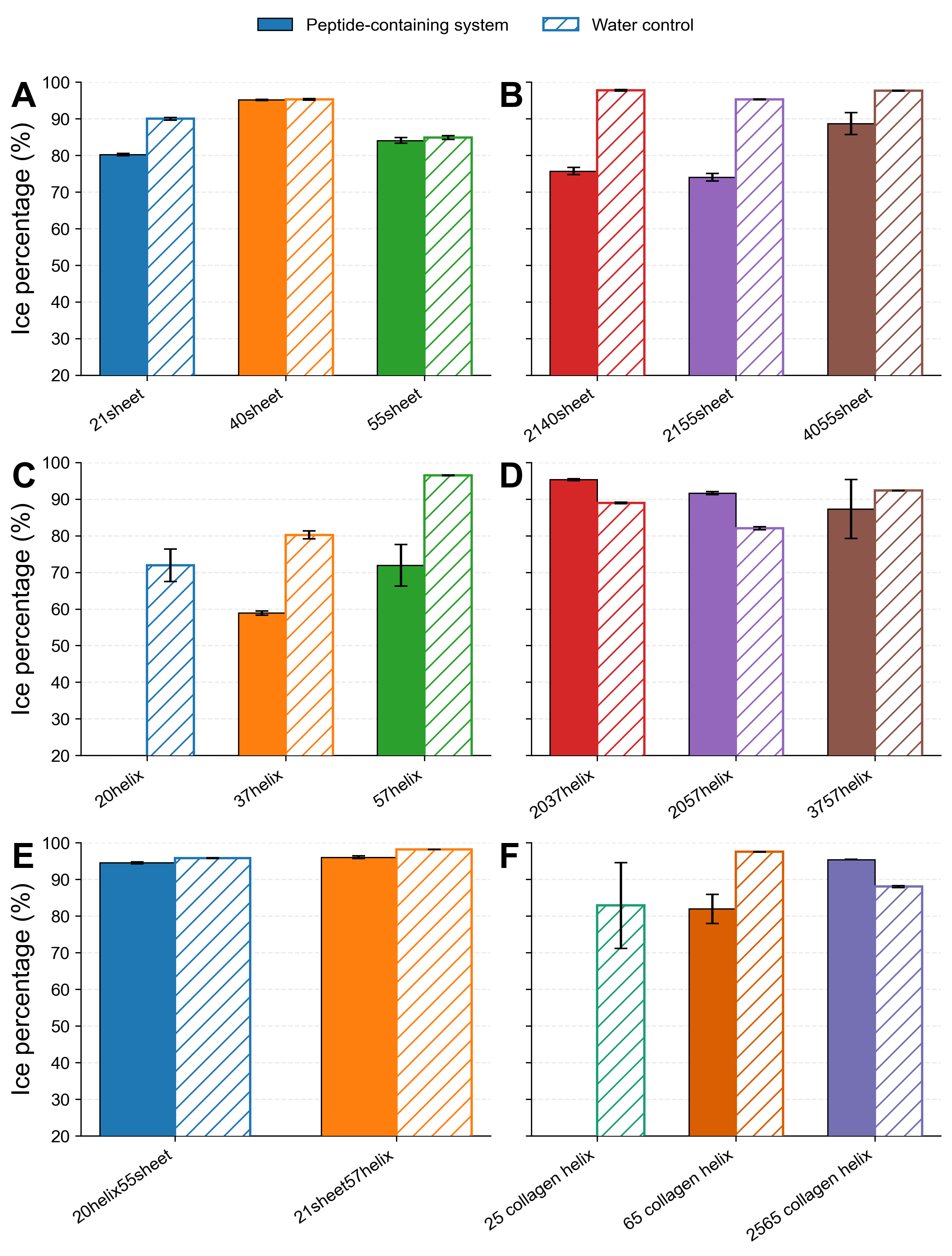

Supplement: Supplementary file 1 [file foods-15-02228-s001.zip › FigureS2.png]

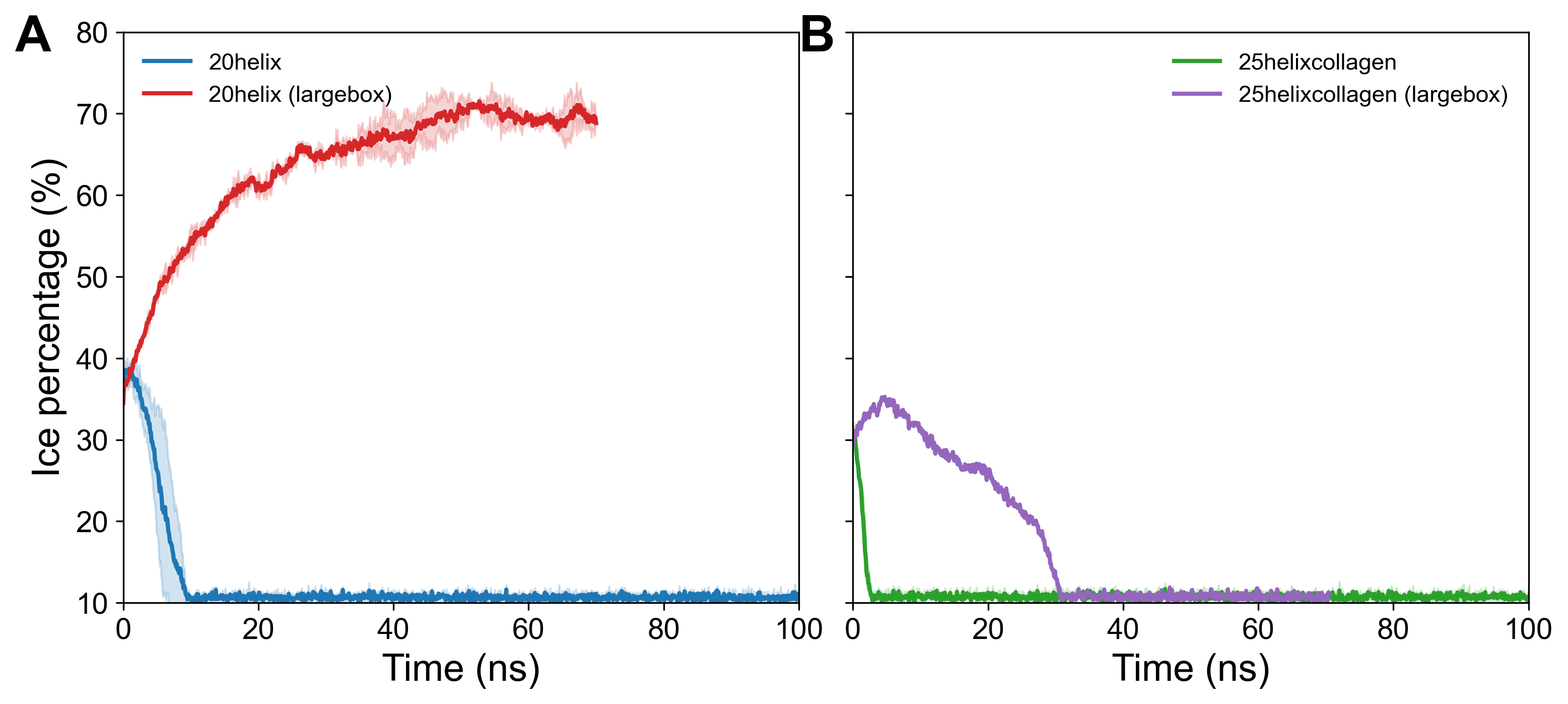

Supplement: Supplementary file 1 [file foods-15-02228-s001.zip › FigureS3.png]

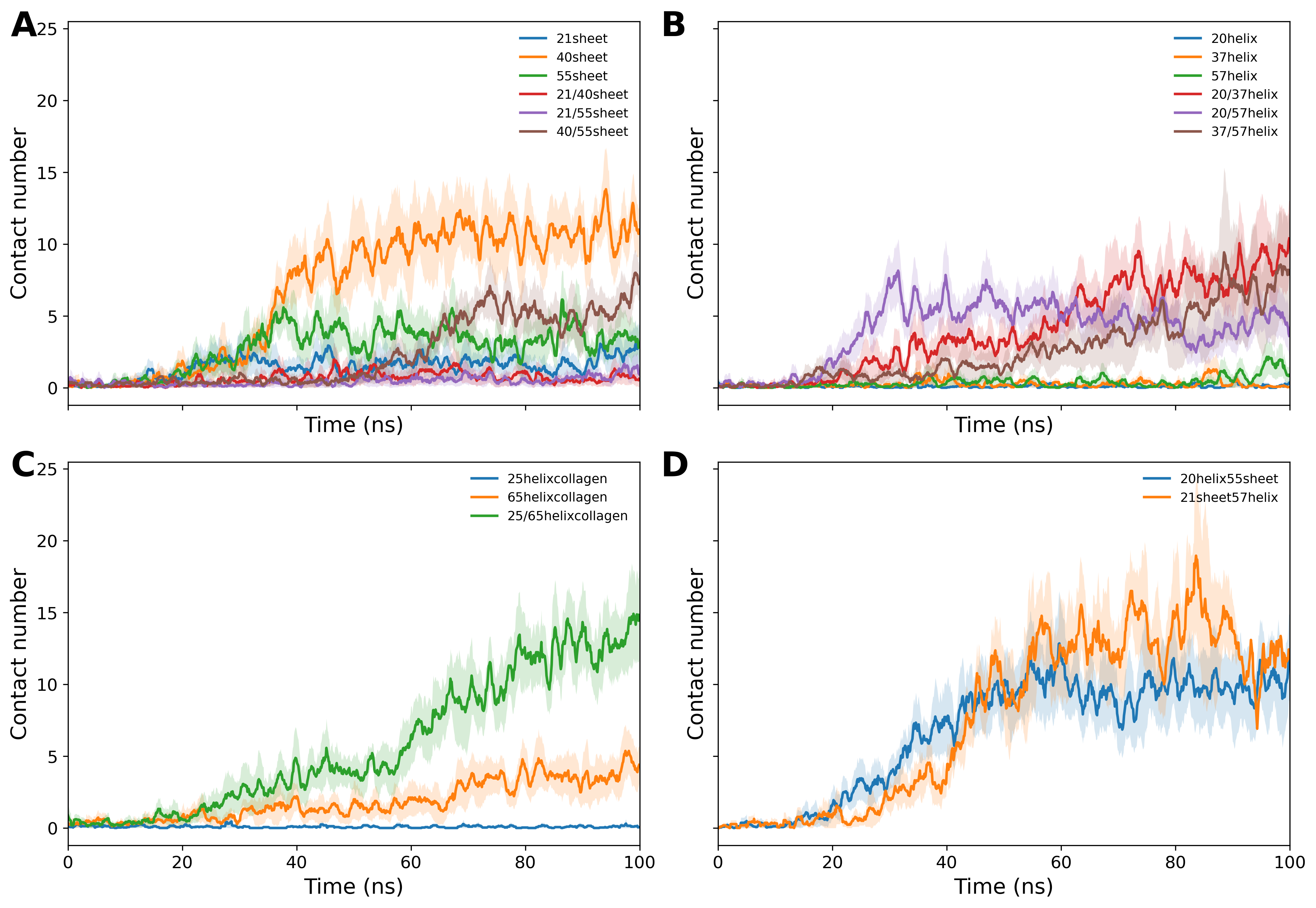

Supplement: Supplementary file 1 [file foods-15-02228-s001.zip › FigureS4.png]
